# Comparison of Outcomes Among Neurovascular Patients Managed in Dedicated Neurological Intensive Care Units vs. General Intensive Care Units

**DOI:** 10.3390/jcm14093090

**Published:** 2025-04-29

**Authors:** Joanna M. Roy, Basel Musmar, Nassos Tziviskos, Saarang Patel, Roberto DeLeon, Ashley Thommana, Shady Mina, Stavropoula I. Tjoumakaris, Michael. Reid Gooch, Robert H. Rosenwasser, Pascal M. Jabbour

**Affiliations:** 1 Department of Neurological Surgery, Thomas Jefferson University Hospital, Philadelphia, PA 19107, USA; joanna.roy@jefferson.edu (J.M.R.); basel.musmar@jefferson.edu (B.M.); shady.mina@students.jefferson.edu (S.M.); stavropoula.tjoumakaris@jefferson.edu (S.I.T.); reid.gooch@jefferson.edu (M.R.G.); robert.rosenwasser@jefferson.edu (R.H.R.); 2 School of Medicine, Rowan-Virtua School of Osteopathic Medicine, Stratford, NJ 08084, USA; nassostziviskos@gmail.com; 3 Seton Hall University, South Orange, NJ 07079, USA; saarangpatel@gmail.com; 4 Syracuse University, Syracuse, NY 13244, USA; rdeleon208503@gmail.com; 5 Pennsylvania State University, Hershey, PA 17033, USA; abt5414@psu.edu

**Keywords:** general intensive care unit, intracerebral hemorrhage, neurocritical care, neurological intensive care unit, stroke

## Abstract

**Background/Objectives**: Patients with neurovascular conditions often require multidisciplinary management to optimize recovery. Our systematic review identifies literature comparing outcomes among neurovascular patients managed at dedicated neurological intensive care units (ICUs) compared to general ICUs. **Methods**: PubMed was searched to identify articles that reported outcomes among patients managed at dedicated neurological ICUs versus general ICUs. Articles that reported outcomes among patients with neurovascular conditions were included. Articles that reported outcomes among patients managed at stroke units were excluded. The Newcastle Ottawa Scale (NOS) was used to assess for risk of bias across individual studies. **Results**: After a title and abstract screen followed by a full-text review, seven studies met criteria for inclusion. These studies reported outcomes among patients managed for intracerebral hemorrhage (ICH), acute ischemic stroke (AIS) and aneurysmal subarachnoid hemorrhage (aSAH). Two studies reported lower mortality, improved functional outcome and reduced costs among patients with ICH who were managed at dedicated neurological ICUs. Among patients with aSAH, only less-severe cases experienced better functional outcome after management at dedicated neurological ICUs. Six out of seven studies were considered high quality. **Conclusions**: Our review highlights the potential benefits of receiving care at dedicated neurological ICUs, as evidenced by lower mortality, improved functional outcome and reduced costs in patients with ICH and low-grade aSAH. However, future research is necessary to clarify whether dedicated neurological ICU care confers significant advantage over general ICUs among patients with AIS and other neurovascular conditions.

## 1. Introduction

Neurovascular conditions such as ischemic stroke, intracerebral hemorrhage (ICH) and aneurysmal subarachnoid hemorrhage (aSAH) necessitate prompt intervention in order to preserve neurological function and optimize recovery [1,2,3]. While some institutions elect to treat patients with these conditions in general intensive care units, many hospitals have specialists and dedicated neurological units for the treatment of such neurovascular conditions [4]. For instance, admission to stroke centers has been associated with lower complications, rates of intracranial hemorrhage and mortality compared to non-stroke centers [5].

Stroke is the second-leading cause of death and third-leading cause of disability worldwide [6]. The number of people who live with a disability after a stroke has increased in recent years, particularly among developing countries [7]. These rates are expected to increase over time, given the growing prevalence of risk factors and comorbid conditions [8]. Intracerebral hemorrhage (ICH) is an acute condition whose incidence correlates positively with increasing age, smoker status and hypertension [9]. Despite recent medical advances, ICH rates have not shown a significant decrease. Deep grey matter structures such as the basal ganglia are often affected by ICH, and prompt medical and/or surgical treatment is required to reduce the risk of poor outcome [10].

Aneurysmal subarachnoid hemorrhage (aSAH) accounts for around 85% of SAH [11]. Similarly to stroke, it has also been shown to have an incidence that correlates with hypertension and smoker status [12]. Location of the aneurysm also plays a significant role, with aneurysms of the posterior circulation being more commonly implicated in aSAH cases [13]. aSAH requires constant monitoring for signs of vasospasm and neurological deterioration [14]. Similarly, patients with intracerebral hemorrhage (ICH) may require early management protocols such as blood pressure control and reversal of anticoagulation [15]. Patients treated at neurological ICUs receive more invasive monitoring and nutritional support compared to patients at general ICUs [16]. However, there is limited knowledge of whether this translates to better post-operative outcomes among patients with neurovascular conditions.

Our systematic review of literature compares outcomes of neurovascular patients treated at neurological ICUs versus general ICUs.

## 2. Materials and Methods

### 2.1. Search Strategy

A comprehensive search of the PubMed database was performed on 24 December 2024 to identify all primary studies comparing ICU and neuro-ICU care for patients with neurovascular conditions. PubMed was queried using the search terms mentioned in Supplemental Table S1.

### 2.2. Inclusion and Exclusion Criteria

Original articles that reported comparative outcomes for neurological ICU care or neurocritical care unit care vs. non-dedicated neurological ICU care were included within this study. Studies that reported outcomes among patients managed with neurointensivists at non-dedicated neurological ICUs were excluded. Additionally, studies reporting outcomes among patients managed at stroke units were excluded. Review articles, letters, non-English articles and articles that reported outcomes in pediatric patients were excluded. Articles that did not include any comparative outcomes between dedicated neuro-ICU/neuro-CCU vs. non-dedicated care were excluded.

### 2.3. Title and Abstract Screening

Search results were screened against title and abstract by two reviewers (AT and RD) based on the appropriate inclusion and exclusion criteria. Each reviewer independently assessed titles and abstracts and was blinded to the other’s decisions to minimize bias. Potential conflicts were resolved by a third reviewer (SP). The Covidence web-based systematic review platform (Covidence, Veritas, Australia) was used to screen articles.

### 2.4. Full Text Review

Full texts were then screened to determine suitability for inclusion in the final review, based on inclusion and exclusion criteria noted above, by two reviewers (AT and RD). Potential conflicts at the full-text screening stage were resolved by a third party (SP).

### 2.5. Data Extraction

The following variables were extracted from each study upon completion of full-text screening: author and year of publication, country, neurovascular subspecialty, patient population, number of patients in each group and outcomes such as functional status (measured using the modified Rankin Scale (mRS) or Glasgow Outcome Scale (GOS), mortality and discharge disposition. Data were manually extracted into a standardized data extraction template on Microsoft Excel (Microsoft Corporation, Redmond, WA, USA).

### 2.6. Qualitative Assessment

The Newcastle Ottawa Scale (NOS) for case-control studies was used to assess for risk of bias across each included study. This scale comprises 3 domains and focuses on selection of participants, comparability and outcome assessment [17]. The selection domain evaluates how cases and controls were selected based on representativeness. Comparability assesses for whether the study design accounts for potential confounders in their analysis. The outcome domain assesses for methods used to determine exposure and outcomes. Each study was independently assessed for risk of bias by two reviewers (J.M.R. and B.M.), following which disagreements were resolved by a third independent review (N.T.). Studies were scored on a scale from 0 to 9, with higher scores indicating lower risk of bias and better quality. Studies with an NOS ≥ 6 were considered high quality, in accordance with the previous literature [18].

### 2.7. Ethical Considerations

Since we utilized deidentified data from previously published studies, this study did not require approval by an Institutional Review Board (IRB).

## 3. Results

### 3.1. Article Search Results

**Figure 1:**
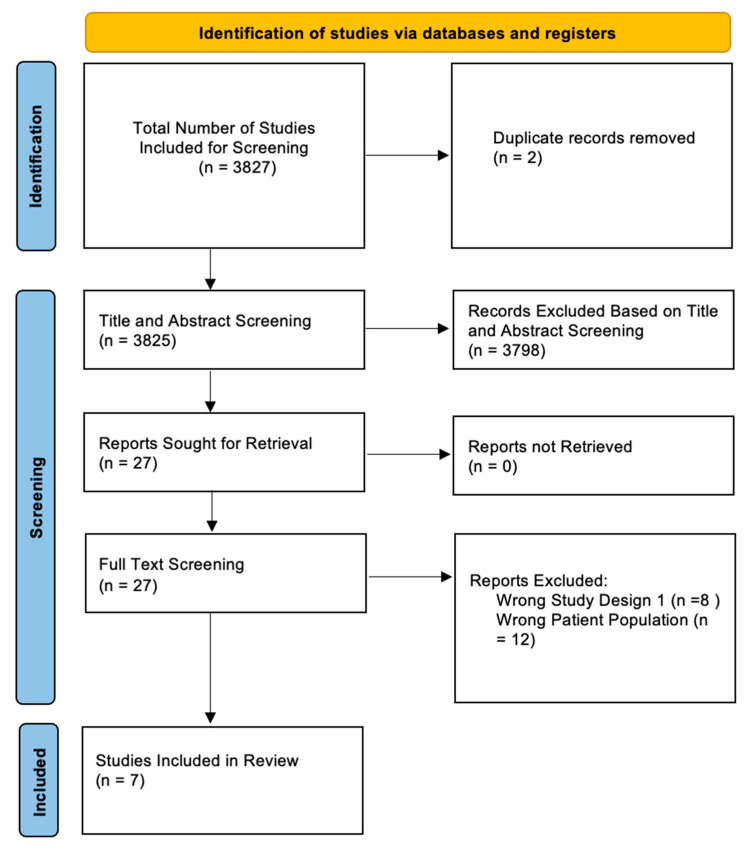
PRISMA diagram depicting selection of studies.

3827 articles were identified through our search string. Two duplicates were removed. A total of 3798 articles were excluded during the title and abstract screen, and 20 articles were excluded during the full text review. Seven studies met the criteria for inclusion in our systematic review (Figure 1).

Three studies reported outcomes in patients with ICH, two studies reported outcomes in aSAH, one study examined outcomes in AIS, and one study reported outcomes in both ICH and AIS. Table 1 lists the characteristics of the included studies.

### 3.2. Intracerebral Hemorrhage

Three studies compared outcomes of ICH patients treated at dedicated neurological ICUs versus non-neurological ICUs. In a single center retrospective study of 128 patients before and after the establishment of a neurological ICU, treatment at neurological ICUs was associated with lower rates of mortality (19% vs. 36%, *p* < 0.05) and better functional outcome (defined as discharge to home or rehabilitation facility, 69% vs. 48%, *p* < 0.05) compared to non-neurological ICUs [20]. These improved metrics, along with shorter durations of hospital stay, translated to reduced costs for patients as well. The second study analyzed data from 1038 patients and 42 participating ICUs across the country. After adjusting for demographics, severity of ICH and ICU and institutional characteristics, admission to non-neurological ICUs was associated with 3.4-fold higher odds of mortality among patients with acute ICH [21]. The third study assessed time to external ventricular drain (EVD) placement and outcomes among 259 patients with spontaneous ICH admitted to critical care resuscitation units (CCRUs), neurological critical care units (NCCUs) or other ICUs [19]. There was no significant difference in time from arrival to EVD placement [170 (106–311) vs. 210 (139–574), *p* = 0.28], hospital length of stay [20 (12–28) vs. 21 (14–31), *p* = 0.47], mortality (25% vs. 25%, *p* = 0.28) or home discharge disposition (19% vs. 24%, *p* = 0.67) among patients treated at CCRUs vs. NCCUs.

### 3.3. Acute Ischemic Stroke

Tran et al. compared outcomes among 128 AIS patients treated at CCRUs and NCCUs [23]. There was no significant difference in mortality or functional independence (mRS ≤ 2) among patients treated at CCRUs vs. NCCUs (16% vs. 30%, *p* = 0.056 and 31% vs. 36%, *p =* 0.59).

### 3.4. Subarachnoid Hemorrhage

Two studies compared outcomes of aSAH patients treated at neurological ICUs versus non-neurological ICUs. In a retrospective study of 755 patients, there was no statistically difference in long-term outcomes, measured using the Glasgow Outcome Scale (GOS), among patients treated at neurosurgical ICUs (n = 456) vs. general ICUs (gICU) (n = 299) (OR: 1.45, 95% CI 0.53–3.95, *p* = 0.46) [24]. Another retrospective study evaluated functional outcome among 151 neurointensivist-managed ICU (NIM-ICU) patients and 83 intensivist-managed ICU (IM-ICU) patients with aSAH [25]. Overall, there was no significant improvement in good functional outcome (mRS 0–2) among patients admitted to NIM-ICUs vs. IM-ICUs. However, a sub-group analysis of patients with less-severe aSAH (defined as Hunt and Kosnik grades I and II) revealed that good functional outcomes were 4.5-fold higher in NIM-ICU patients (odds ratio = 4.54, 95% CI 1.08–22.17, *p* = 0.04). NIM-ICU patients also had shorter hospital lengths of stay compared to IM-ICU patients; however, they remained in the ICU longer than IM-ICU patients.

### 3.5. Intracerebral Hemorrhage and Acute Ischemic Stroke

Lott et al. utilized a nationwide database to analyze outcomes among patients treated at general ICUs, ideal specialty ICUs and non-ideal specialty ICUs [22]. Ideal specialty ICUs for patients with ICH and AIS were defined as neurological ICUs. After adjusting for baseline demographics and hospital characteristics, they reported no difference in mortality for ICH patients admitted to neurological ICUs compared to patients admitted to general ICUs or non-ideal specialty ICUs. In patients with AIS, admission to non-ideal specialty ICUs was associated with 1.41-fold higher odds of mortality compared to general ICUs. Admission to neurological ICUs did not confer a significant difference in mortality.

### 3.6. Risk of Bias Assessment

Most studies were considered high quality based on the NOS. Table 2 presents scores for each individual study. All studies included hospital controls and scored three points for selection. Six studies adjusted for confounders such as age, sex and clinical presentation through a logistic regression model. However, none of the studies in our review provided details on non-response rates or the distribution of missing data among cases and controls.

## 4. Discussion

### 4.1. Key Results

Our review identified two studies reporting lower mortality, better functional outcome and reduced costs for ICH patients managed in dedicated neurological ICUs. Among aSAH patients, only those with less-severe cases had improved functional outcomes at dedicated neurological ICUs.

### 4.2. Research Background

Neurocritical care has been associated with favorable functional outcome, length of stay and discharge disposition among patients undergoing treatment for neurovascular conditions. This has been attributed to close monitoring of patients, which allows for early recognition of neurological deterioration. For instance, patients admitted to neurological ICUs are more likely to undergo invasive intracranial or hemodynamic monitoring to prevent hypotension and hypovolemia, in addition to early tracheostomy to prevent aspiration and undernutrition [16]. This suggests that positive outcomes following admission to neurological ICUs may stem from the initiation of more safety-related protocols to minimize the risk of deterioration. Neurological ICUs often comprise a multi-disciplinary team of neurointensivists, nursing staff and advanced practice nurses (APNs) with specialized training in neurocritical care [26]. Admission to dedicated neurological ICUs has been associated with cost-effective care due to shorter length of stay, better discharge disposition and lower mortality rates among patients with traumatic brain injury [27].

### 4.3. Review of the Literature

Our review of the literature identified two studies that reported lower rates of mortality, improved functional outcome and reduced costs among ICH patients who were managed at neurological ICUs compared to non-neurological ICUs. Improved outcomes in these studies were attributed to the ability of neurological ICUs to provide constant monitoring of patients and identify early signs of neurological deterioration. ICH accounts for about 10–20% cases of all strokes and requires prompt management to minimize the risk of severe disability or death [28,29]. Early triage also helps identify candidates for surgical intervention [30]. However, it is worth noting that resources to establish a dedicated neurological ICU may not be available at all centers. Burns et al. reported improved quality of care indicated by more effective dysphagia screening and blood pressure control after providing neurocritical care services to patients with ICH despite a dedicated neurological ICU [31]. This suggests the possibility of introducing the neurocritical care model through a team of trained neurointensivists at resource-limited hospitals where a neurological ICU cannot be established. The third study compared outcomes among ICH patients who arrived at CCRUs, NCCUs and non-dedicated ICUs [19]. They reported comparable outcomes among patients treated at CCRUs to those treated at NCCUs. The CCRU team comprised intensivists and advanced-practice providers (APPs) who were trained to provide initial resuscitation, blood pressure management and support for EVD placement. In a similar study that analyzed the impact of CCRUs in patients with AIS with large-vessel occlusion, it was found that CCRUs provided comparable care to NCCUs [23]. These findings suggest that appropriate training of providers may be adequate to treat ICH patients in institutions that do not have the infrastructure and essential resources to form a dedicated neurological care facility.

Among studies that reported outcomes among patients admitted with aSAH, only patients with less-severe aSAH demonstrated significant improvement in functional outcome after admission at the neurological ICU compared to a non-neurological ICU [25]. Mielke et al. reported no significant difference in outcomes of aSAH patients treated at neurosurgical ICUs versus general ICUs [24]. Lack of significant difference was attributed to the importance of providing general intensive care through respiratory therapy or sedation, which outweighed benefits due to early detection and management of vasospasm or hydrocephalus in aSAH patients. They concluded that the presence of general intensive care could supplement the knowledge of a neurosurgeon in providing aSAH care. The difference in functional outcome witnessed between lower-acuity patients treated in neurological and non-neurological ICUs may be due to the potential nuances in the treatment of mild aSAH that would be less familiar to providers who are not specialized in neurocritical care. The interventions necessary for aSAH patients in more severe conditions may have more well-known and strictly followed protocols in each institution, whereas relatively milder conditions may be routinely under-managed in units that are not specifically trained to do so. Additionally, complications of aSAH involve organ systems beyond the nervous systems [32]. As such, more severe aSAH may necessitate further interdisciplinary involvement, effectively equalizing the level of treatment between dedicated and general ICUs. Furthermore, the overall degree to which a patient with severe aSAH can be rescued is more likely to be poor, and aggressive surgical intervention in Hunt and Hess grade IV and V aneurysms has been associated with high rates of morbidity and mortality [33,34]. In such cases, the lack of significant difference between neurological ICUs and non-neurological ICUs could be attributed to severity of presentation. The previous literature has reported reduced lengths of stay and mortality rates and improved discharge disposition from the co-management of aSAH patients with a multidisciplinary team comprising neurointensivists and neurosurgeons [35,36].

In patients with acute ischemic stroke, Tran et al.’s study found that there was no significant difference in function or mortality between AIS patients treated in NCCUs and CCRUs [23]. In a similar vein, a different paper showed that the mortality rate of AIS patients being administered care in the neurological ICU setting did not differ significantly from those admitted to the general ICU; however, an increase in mortality was seen in those admitted to non-ideal specialty ICUs [22]. Apart from cases where a patient is eligible for neurointerventional treatment, or surgery for cerebral edema is warranted, early management of AIS is mostly medical [37]. According to the previous literature, eligibility for mechanical thrombectomy in large-vessel occlusions has been estimated to range between 10 and 30%, with another study noting that only 14% of potentially eligible patients received mechanical thrombectomy [38,39]. This is in contrast to aSAH, where aneurysms are commonly treated through endovascular means or surgically clipped [40]. It may be the case that for conditions where neurosurgical intervention is less involved, such as in the majority of AIS patients, similar to poor-grade Hunt and Hess aSAH patients previously mentioned, general ICUs retain sufficient means to treat patients to the level that neurological ICUs do.

### 4.4. Future Research Directions

Our study highlights the need for prospective studies that assess outcomes among patients treated at dedicated neurological ICUs versus general ICUs for neurovascular conditions. Dedicated neurological ICUs may not be available at resource-limited centers. Future research could aim to analyze the feasibility of neurological ICUs in resource-limited settings. Low-income and low-middle-income countries lack resources such as ICU beds, neuroimaging facilities and clinical laboratories, in addition to trained neurointensivists or neurosurgeons to manage emergent conditions [41]. Thus, guidelines developed for neurological ICUs may not be applicable in resource-limited areas due to limited infrastructure and higher rates of morbidity compared to resource-rich environments [42]. Studies that assess the cost-effectiveness of managing neurovascular conditions at dedicated neurological ICUs compared to general ICUs would also provide additional information on the benefits of dedicated neurological ICU care. Developing risk stratification measures to identify which patients would benefit from care at dedicated neurological ICUs could also improve healthcare resource utilization. Stroke units are a potential alternative to providing multidisciplinary care for patients with neurovascular conditions. Admission to stroke units has been associated with improved functional outcome on discharge compared to admission to general neurology wards [43]. In patients with low-grade, non-aneurysmal SAH, admission to stroke units had similar rates of mortality, functional outcome and post-operative complications compared to admission to neurological ICUs [44]. Stroke units are a cost-effective alternative to ICU admissions in developing countries and allow for better healthcare resource allocation of ICU beds to neurocritical care patients. However, future research is necessary to identify which patients would benefit from admission to stroke units compared to dedicated neurological ICUs [45].

### 4.5. Limitations

Our systematic review is subject to limitations. First, we restricted our inclusion criteria to comparative studies reporting outcomes among patients treated at neurological ICUs and non-neurological ICUs. We also chose to exclude stroke units from our review. This may have reduced the sample size of studies included. We also restricted our search to a single database and articles published in the English language. Due to the heterogeneity of study design, patient populations and outcomes across individual studies, we were unable to perform a meta-analysis. We would also like to acknowledge the potential for selection bias, since patients admitted at dedicated neurological ICUs may have been selected based on their underlying clinical severity or institutional protocols that varied across studies. Results in each study may have also been subject to bias due to unmeasured confounders such as patient demographics, comorbidities, differences in hospital infrastructure, staffing protocols, admission protocols and provider experience. Most studies in our cohort described short-term outcomes such as length of stay and functional outcome on discharge. There is limited knowledge of the impact of dedicated neurological ICU care on long-term outcomes such as quality of life. Our risk-of-bias assessment revealed that most studies did not account for the proportion of missing cases or controls. This may have introduced bias.

## 5. Conclusions

Our review highlights the potential benefits of dedicated neurological ICU care among patients with neurovascular conditions. While evidence suggests improved functional outcome and lower mortality among patients with ICH and low-grade aSAH, further research is needed to determine whether neurological ICU care provides significant advantages over general ICU care for AIS and other neurovascular conditions.

## Figures and Tables

**Table 1:** Characteristics of included studies.

Author	Year	Country	Pathology	Comparison
Tran et al. [19]	2021	USA	ICH	NCCU vs. CCRU vs. other ICUs
Mirski et al. [20]	2001	USA	ICH	Neurological ICU vs. general ICU (surgical or medical ICUs)
Diringer et al. [21]	2001	Germany	ICH	Neurosurgical ICU vs. general ICU
Lott et al. [22]	2009	USA	AIS and ICH	General ICU vs. neurological ICU vs. non-ideal specialty ICU
Tran et al. [23]	2020	USA	AIS	NCCU vs. CCRU vs. other ICU
Mielke et al. [24]	2019	Germany	aSAH	Neurosurgical ICU vs. general ICU
Egawa et al. [25]	2016	Japan	aSAH	Intensivist-managed ICU vs. neurointensivist-managed ICU

Abbreviations: AIS: acute ischemic stroke, aSAH: aneurysmal subarachnoid hemorrhage, CCRU: critical care resuscitation units, ICU: intensive care unit.

**Table 2:** Risk of bias assessment using the Newcastle Ottawa Scale.

Author, Year	Selection	Comparability	Exposure	Total
Tran et al. [19]	3	2	2	7
Mirski et al. [20]	3	0	2	5
Diringer et al. [21]	3	2	2	7
Lott et al. [22]	3	2	2	7
Tran et al. [23]	3	2	2	7
Mielke et al. [24]	3	2	2	7
Egawa et al. [25]	3	2	2	7

## Data Availability

Data will be made available upon reasonable request from the corresponding author.

## Supplementary Materials

The following supporting information can be downloaded at: https://www.mdpi.com/article/10.3390/jcm14093090/s1, Table S1: PubMed search string.

## Abbreviations

The following abbreviations are used in this manuscript:

AIS	acute ischemic stroke
ICH	intracerebral hemorrhage
ICU	intensive care unit
