# Supplementary material for: Comparison of Outcomes Among Neurovascular Patients Managed in Dedicated Neurological Intensive Care Units vs. General Intensive Care Units
Source: J Clin Med. 2025 Apr 29;14(9):3090. doi: 10.3390/jcm14093090 (PMC12072713; doi:10.3390/jcm14093090)
Supplement: Supplementary file 1 [file jcm-14-03090-s001.zip › jcm-3539256-supplementary.pdf]

Table S1: PubMed search string

	(((((((mortality[Title/Abstract]) OR (morbidity[Title/Abstract])) OR (prognosis[Title/Abstract])) OR (discharge[Title/Abstract])) OR (outcome[Title/Abstract])) OR (survival[Title/Abstract])) OR (death[Title/Abstract])) AND (((((((neurovascular[Title/Abstract]) OR (stroke[Title/Abstract])) OR (intracranial hemorrhage[Title/Abstract])) OR (intracerebral hemorrhage[Title/Abstract])) OR (ischemic stroke[Title/Abstract])) OR (subarachnoid hemorrhage[Title/Abstract])) AND ((((intensive care unit*[Title/Abstract]) OR (critical care[Title/Abstract])) OR (critical nursing[Title/Abstract])) OR (critical care unit[Title/Abstract])))
